# Crosstalk among lncRNAs, microRNAs and mRNAs in the muscle ‘degradome’ of rainbow trout

**DOI:** 10.1038/s41598-018-26753-2

**Published:** 2018-05-30

**Authors:** Bam Paneru, Ali Ali, Rafet Al-Tobasei, Brett Kenney, Mohamed Salem

**Affiliations:** 1 Department of Biology and Molecular Biosciences Program, Middle Tennessee State University, Murfreesboro, TN 37132 USA; 2 Computational Science Program, Middle Tennessee State University, Murfreesboro, TN 37132 USA; 3 Department of Biostatistics, University of Alabama at Birmingham, Birmingham, AL 35294-0022 USA; 4 Division of Animal and Nutritional Science, West Virginia University, Morgantown, 26506-6108 West Virginia USA

## Abstract

In fish, protein-coding and noncoding genes involved in muscle atrophy are not fully characterized. In this study, we characterized coding and noncoding genes involved in gonadogenesis-associated muscle atrophy, and investigated the potential functional interplay between these genes. Using RNA-Seq, we compared expression pattern of mRNAs, long noncoding RNAs (lncRNAs) and microRNAs of atrophying skeletal muscle from gravid females and control skeletal muscle from age-matched sterile individuals. A total of 852 mRNAs, 1,160 lncRNAs and 28 microRNAs were differentially expressed (DE) between the two groups. Muscle atrophy appears to be mediated by many genes encoding ubiquitin-proteasome system, autophagy related proteases, lysosomal proteases and transcription factors. Transcripts encoding atrogin-1 and mir-29 showed exceptional high expression in atrophying muscle, suggesting an important role in bulk muscle proteolysis. DE genes were co-localized in the genome with strong expression correlation, and they exhibited extensive ‘lncRNA-mRNA’, ‘lncRNA-microRNA’, ‘mRNA-microRNA’ and ‘lncRNA-protein’ physical interactions. DE genes exhibiting potential functional interactions comprised the highly correlated ‘lncRNA-mRNA-microRNA’ gene network described as ‘degradome’. This study pinpoints extensive coding and noncoding RNA interactions during muscle atrophy in fish, and provides valuable resources for future mechanistic studies.

## Introduction

Sexual maturation, starvation and several pathological conditions negatively affect muscle mass and fillet quality attributes^1–3^. Improving growth performance and fillet quality by reducing protein turnover requires an understanding of muscle proteolysis, *in vivo*. Previously, several studies have identified protein-coding genes associated with skeletal muscle atrophy in fish^1–3^. A previous microarray study identified about 200 protein-coding genes that were differentially expressed (DE) during sexual maturation and associated with muscle atrophy in trout^1^. This study also found upregulated expression of catheptic and collagenase proteolytic pathways during muscle atrophy. Additionally, activation of calpains and the 28S proteasome subunit during starvation induced skeletal muscle atrophy previously was observed^4^. Albeit, some of the previous findings are inconsistent and do not provide a comprehensive set of protein-coding genes associated with muscle atrophy. As an example, some studies have reported downregulation of ubiquitin-proteasome system during atrophy^1^, while others have reported its upregulation^5,6^. These studies either investigated a single protein-coding gene^5,6^ or limited sets of protein-coding genes^1^ due to lack of a holistic approach. Moreover, none of the previous studies investigated the role of microRNAs and long noncoding RNAs (lncRNAs) in trout muscle atrophy. A more robust approach is needed to discover all potential candidate genes involved in muscle atrophy.

MicroRNAs bind 3′ -UTR of mRNA that leads to downregulation of the gene by various mechanisms such as translation suppression^7^, target mRNA cleavage^8^ and deadenylation^9^. There is evidence that a single microRNA can regulate hundreds of genes; and, at the same time, a single gene can be regulated by hundreds of microRNAs^10^. MicroRNAs are known to regulate muscle proteolysis and muscle atrophy in different mammalian species^11^. For example, mir-486 regulates disease-related muscle atrophy in mice by regulating FOXO1 transcription factor^12^. MicroRNA, mir-182 indirectly regulates the expression of key muscle atrophy genes including atrogin-1, cathepsins and autophagy related genes by targeting transcription factor FOXO3^13^. Muscle specific microRNA, mir-1, regulates dexamethasone mediated muscle atrophy by targeting heat shock protein 70 (HSP70)^14^. However, microRNAs that regulate muscle atrophy in salmonids have not been previously investigated.

LncRNAs are a recently discovered class of noncoding RNAs with critical gene regulatory roles^15^. LncRNAs are known to regulate genes by direct interaction with microRNAs, mRNAs and proteins. Several lncRNAs bind microRNAs by sequence complementarity, and this interaction leads to cellular sequestration of microRNA (sponge effect) and lncRNA-mRNA competition for microRNA binding^16^. For example, lncRNA H19 binds and sponges away let-7 family microRNAs from repressing its protein-coding targets^17^. Similarly, muscle specific lncRNA, linc-MD1, competes with MAML1 and MEF2C to bind microRNAs mir-135 and mir-133, respectively^18^. LncRNA, MALAT1, modulates mir-133 mediated downregulation of serum response factor (SRF) by sharing mir-133 binding site^19^. LncRNAs can directly bind or physically interact with mRNA leading to mRNA decay^20^ and translation suppression^21^. Some lncRNAs hybridize with the 3′ UTR of target mRNA and facilitate Staufen-1 mediated mRNA decay^20^. On the other hand, lincRNA-p21 directly binds *JUNB* and *CTNNB1* mRNAs and suppresses their translation^21^. LncRNA’s physical interaction with proteins modulates the stability^22^, cellular availability (sequestration)^23^, activity^24^ and cellular localization^24^ of proteins. For example, lncRNA, UPAT1, binds to UHRF1 protein and interferes with its ubiquitination and subsequent degradation^22^. LncRNA, MALAT1, binds to SR splicing factors and regulates their phosphorylation and hence cellular localization^24^. Both ‘lncRNA-microRNA’ and ‘lncRNA-protein-coding genes’ interactions are known to regulate development^18^, disease^25^ and cancers^26,27^; however, their involvement in skeletal muscle atrophy remains unknown.

To identify coding and noncoding genes involved in muscle atrophy associated with sexual maturation, we sequenced mRNAs, lncRNAs, and microRNAs from atrophying skeletal muscle of gravid fish and normal skeletal muscle of sterile fish. We subsequently performed differential gene expression of the two groups. In addition, we investigated functional interactions between DE lncRNAs, microRNAs and protein-coding genes in terms of expression correlation, genome co-localization and physical interaction to investigate gene-regulatory circuits during muscle atrophy. This study provides the first genome-wide lncRNA-mRNA-microRNA interaction network describing fish muscle degradation, and defining these interactions will clarify how energetic demand at sexual maturation triggers skeletal muscle atrophy.

## Result and Discussion

### Characteristics of atrophying and normal skeletal muscle

**Figure 1:**
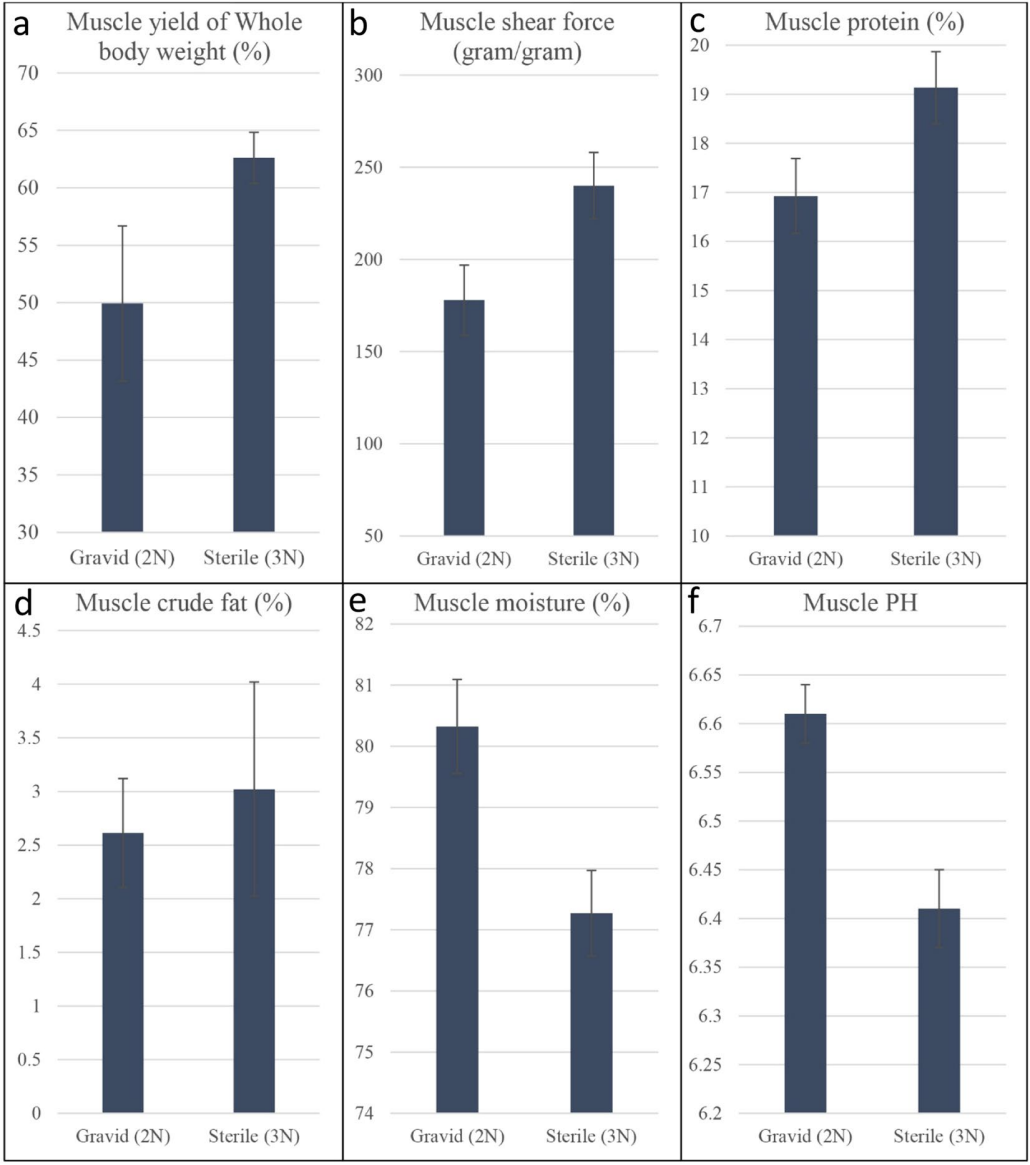
Comparison of different muscle phenotypes between atrophying skeletal muscle from gravid diploid (2N) fish and non-atrophying muscle from sterile triploid (3N) fish. Bar graph shows mean muscle yield (% of whole body weight) (**a**), muscle shear force (gram/gram) (**b**), muscle protein content (%) (**c**), muscle crude fat content (%) (**d**), muscle moisture content (%) (**e**) and muscle PH (**F**) of five sterile and five fertile fish at the age of spawning. Error bar represent standard error.

Differential expression of coding and noncoding genes was performed in atrophying skeletal muscle from diploid (2N), gravid fish in comparison to non-atrophying muscle of sterile triploid (3N) fish. This same set of skeletal muscle samples was used in our laboratory for several previous studies^1,28,29^. Compared to sterile fish, fertile females yielded less separable muscle per whole body weight (49.9% ± 6.7% vs 62.6% ± 2.2%, p = 0.01), muscle protein (16.9% ± 0.7% vs ~19.1% ± 0.7%, p = 0.01) and muscle shear force (178 ± 19 gram/gram vs 240 ± 18 gram/gram, p = 0.01). On the other hand, atrophied skeletal muscle from 2N females had a higher moisture content (80.3% ± 0.7% vs 77.2% ± 0.6%) and pH (6.61 ± 0.03 vs 6.41 ± 0.04) (Fig. 1). Atrophied muscle also had numerically lower crude fat content than normal muscle, but the difference was not statistically significant (p = 0.30). These textural and compositional difference between two groups of muscle result from extensive muscle atrophy in gravid fish triggered by the energetic demand of sexual maturation.

### Differential expression of mRNAs, lncRNAs and microRNAs in atrophying muscle

**Figure 2:**
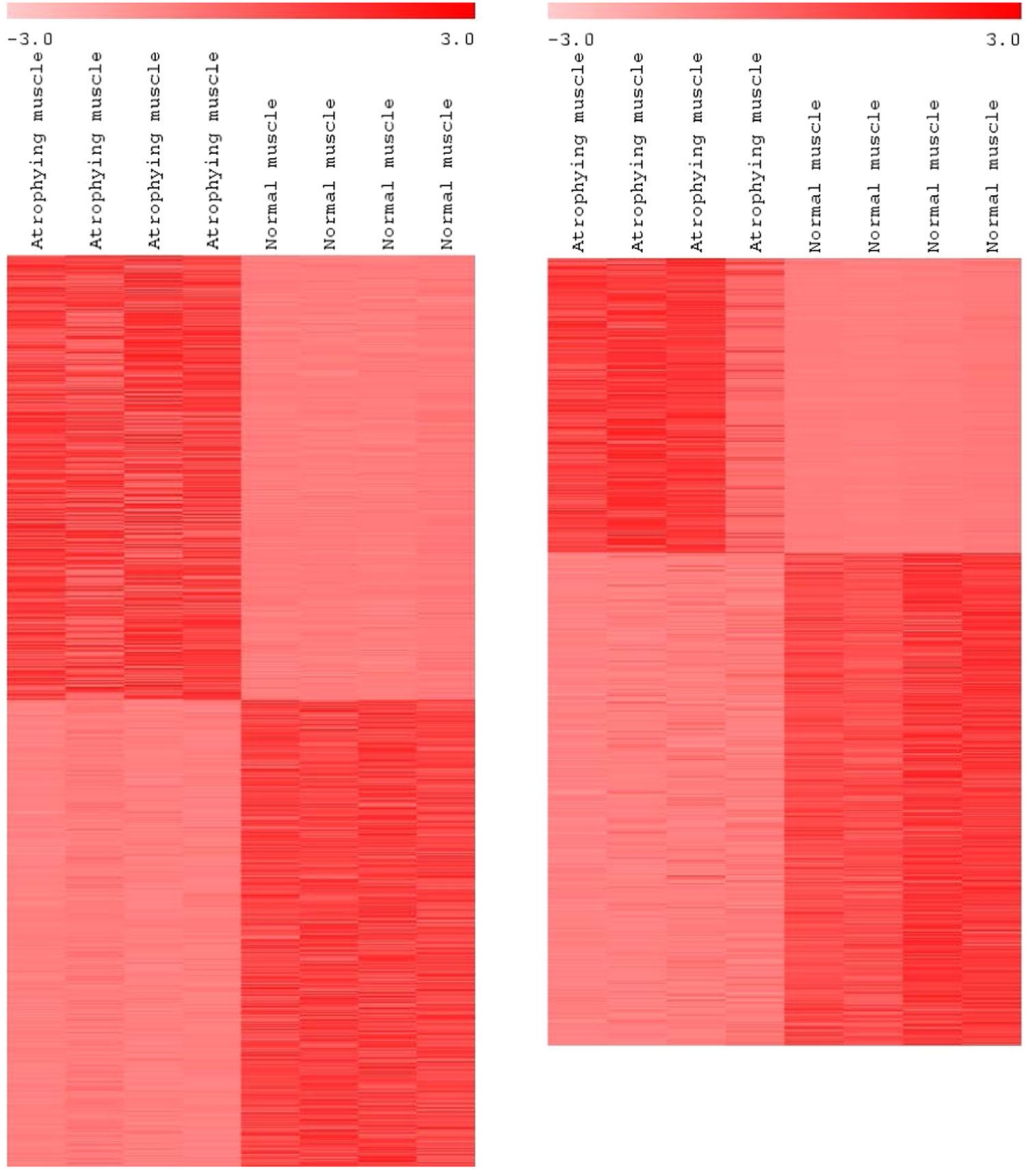
Heat map of DE lncRNAs (left) and protein-coding genes (right) between atrophying muscle of gravid fish and non-atrophying muscle of sterile fish. Value of color limit represents normalized expression values (Z scores). Fold change in gene expression was considered significant at: FDR-p-value < 0.01, fold change: >3 or <−3. Darker red and lighter red colors represent higher and lower level of expression, respectively. Transcript annotations are provided in Supplementary dataset 2.

To identify genes likely involved in skeletal muscle atrophy during sexual maturation, we performed deep lncRNA, mRNA and microRNA sequencing, and quantified DE genes between atrophying skeletal muscle of gravid fish and non-atrophying skeletal muscle from sterile fish. A total of 852 mRNAs, 1,160 lncRNAs and 28 microRNAs were DE between these two groups (FDR-p-value < 0.01, fold change: > 3 or <−3) (Fig. 2, Table 1 and Supplementary dataset 1). A total of 1,025 transcripts (352 mRNAs, 661 lncRNAs and 12 microRNAs) were upregulated and 1,015 transcripts (500 mRNAs, 499 lncRNAs and 16 microRNAs) were downregulated in atrophying muscle. Real time PCR validation of 4 transcripts from each DE list of lncRNAs, microRNAs and mRNAs is provided in Supplementary dataset 2. Previously, a microarray based approach performed on the same set of muscle samples identified only 82 upregulated and 120 downregulated protein-coding genes^1^, suggesting identification, in this study, of a large number of additional candidate genes involved in muscle atrophy. DE protein-coding genes, lncRNAs and microRNAs are described in separate sections hereafter.

**Table 1:** DE microRNAs between atrophying muscle of gravid fish and normal skeletal muscle of sterile fish.

MicroRNA	Fold change	FDR p-value correction
let-7j	−1056.3	0.00069
mir-7641-1	−9.3	0.00002
mir-2187	−8.1	0.008
mir-7551	−6.8	0.00878
mir-181a-2	−5.5	0.00211
mir-1a-2	−5.2	0.006
mir-7641	−4.7	0.00172
mir-1386	−3.8	0.00603
let-7c-1	−3.8	0.00211
let-7a-3	−3.6	0.007
mir-203b	−3.6	0.008
mir-1–3	−3.6	0.009
mir-148a	−3.5	0.00069
mir-125b-1	−3.3	0.00256
mir-15b	−3.2	0.00603
mir-133a-1	−3	0.0062
mir-132b	3	0.008
let-7d	3.3	0.00977
mir-146a	3.4	0.0061
mir-132-1	3.7	0.0072
mir-29c-3p	4.2	0.00005
mir-29c	5.1	0.00001
let-7	5.2	0.00069
mir-457b	5.7	0.00025
mir-29b-2	7	0.000002
mir-29b	7.6	0.000154
mir-29b-1	9.6	0.00001
mir-29a	11.7	0.00013

Positive and negative value of fold change represent upregulation and downregulation respectively in atrophying skeletal muscle of gravid fish. Fold change was considered significant at cutoff: > 3 or <−3, FDR-p-value < 0.01. Several isoforms of let-7 were downregulated and several isoforms of mir-29 were upregulated.

### Protein-coding genes

Many genes that promote proteolysis were significantly upregulated in atrophying skeletal muscle. At least 37 genes involved in protein ubiquitination, 22 genes involved in autophagy-related proteolysis, and 15 lysosomal and other proteases (cathepsin D, cathepsin B, cathepsin L and cathepsin Z) showed upregulation in atrophying muscle (Table 2 and Supplementary dataset 1). On the other hand, genes that negatively regulate the ubiquitin-proteasome system (ubiquitin carboxyl-terminal hydrolase 10, ubiquitin-like domain-containing CTD phosphatase 1 and uridine-cytidine kinase 2) and autophagy (CDGSH iron-sulfur domain-containing protein 2) were downregulated. Amino acid and fat biosynthetic genes were downregulated while genes involved in amino acid catabolism and transport were highly upregulated (Supplementary dataset 1). Similarly, genes associated with muscle sarcomere and extracellular matrix were downregulated, consistent with the loss of muscle mass and shear force during atrophy. As an example, 47 collagen-related genes and 24 non-collagen, extracellular matrix protein genes were significantly downregulated. A previous study also showed similar expression pattern of genes involved in protein ubiquitination and associated with the autophagy-lysosome system, extracellular matrix and sarcomere structure during muscle atrophy in mammals^30^. At least 53 transcription factors (TFs) or transcription regulators were also DE; of these transcription factors or regulators, 28 were upregulated and 25 were downregulated (Supplementary dataset 1). While the proteolytic role for the majority of the TFs was unknown, some transcription regulators, such as zinc finger and BTB domain-containing protein 16 and ddb1- and cul4-associated factor 6, had known function in protein catabolism. On the other hand, development related TFs like myoD were downregulated. These findings suggest that muscle atrophy is triggered by upregulation of proteolytic and catabolic genes with concomitant downregulation of muscle sarcomere, extracellular matrix, muscle development and biosynthetic genes.

**Table 2:** Selected proteolytic genes highly upregulated in atrophying skeletal muscle of gravid female rainbow trout relative to non-atrophying muscle of same-aged sterile rainbow trout.

DE mRNA ID	DE mRNA name	Fold change	FDR p-value correction
**Genes involved in ubiquitin-mediated protein degradation**			
GSONMT00016768001	f-box only protein 32/fbxo32/atrogin-1	377.71	6.909E-16
GSONMT00031929001	f-box only protein 32/fbxo32/atrogin-1	152.44	1.629E-15
GSONMT00049913001	kelch-like protein 38-like	54.13	3.128E-06
GSONMT00006333001	kelch-like protein 33-like	37.99	6.822E-05
GSONMT00021608001	zinc finger and btb domain-containing protein 16-a-like	35.37	2.365E-08
GSONMT00076944001	tribbles homolog 2	9.59	0.0002851
GSONMT00082158001	otu domain-containing protein 1	9.40	1.475E-06
GSONMT00079892001	tumor protein p53-inducible nuclear protein 2	7.05	3.306E-07
GSONMT00000505001	thioredoxin-interacting protein	6.98	2.585E-05
TCONS_00090611	E3 ubiquitin-protein ligase HERC2-like	6.76	0.0059419
TCONS_00080006	speckle-type POZ protein	6.55	0.0006765
GSONMT00074639001	ubiquitin carboxyl-terminal hydrolase 25-like isoform x2	6.52	2.04E-06
GSONMT00036946001	ubiquitin-conjugating enzyme e2 g1	6.37	0.0007905
GSONMT00064758001	e3 ubiquitin-protein ligase znrf2	6.24	0.0043357
GSONMT00009231001	ddb1- and cul4-associated factor 6-like isoform x4	6.18	2.024E-05
**Lysosomal proteases**			
GSONMT00080266001	cathepsin b	4.96	0.0001879
GSONMT00063049001	cathepsin L1	8.46	3.032E-07
GSONMT00049973001	cathepsin z precursor	3.49	0.0033306
TCONS_00051616	cathepsin D	3.89	4.219E-05
**Autophagy related proteases**			
GSONMT00065684001	protein soga3-like isoform x3	63.81	2.426E-09
GSONMT00024835001	transmembrane protease serine 5-like	21.41	0.0006682
GSONMT00078909001	serine threonine-protein kinase ulk2-like isoform	12.20	2.651E-05
GSONMT00059371001	cysteine protease atg4b	12.07	4.249E-06
GSONMT00069267001	autophagy-related protein 9a-like isoform x1	11.17	0.0001673
GSONMT00012216001	gamma-aminobutyric acid receptor-associated 1	9.65	6.69E-07
GSONMT00067581001	serine threonine-protein kinase ulk2	9.31	0.0058011
GSONMT00031082001	autophagy-related protein 2 homolog a-like	7.12	0.0002591
GSONMT00037970001	autophagy-related protein 2 homolog b-like	5.97	0.0012979
GSONMT00075003001	beclin 1-associated autophagy-related key regulator	5.77	0.0022351

Fold change was considered significant at cutoff: > 3 or <−3, FDR-p-value < 0.01.

The ubiquitin proteasome system appeared to be the major proteolytic system governing muscle atrophy. F-box only protein 32 (FBXO32) (atrogin-1), an E3 ubiquitin ligase, was the most highly upregulated genes in atrophying muscle suggesting that it might be the major player of muscle proteolysis during atrophy. Atrogin-1 genes transcripts, GSONMT00016768001 and GSONMT00031929001, exhibited 378- and 152-fold upregulation, respectively (Table 2). Their expression was validated by real time PCR (Supplementary dataset 2). Overexpression of atrogin-1 during starvation induced skeletal muscle atrophy has been reported previously in rainbow trout^5^, Atlantic salmon^6^ and mammals^31^.

**Figure 3:**
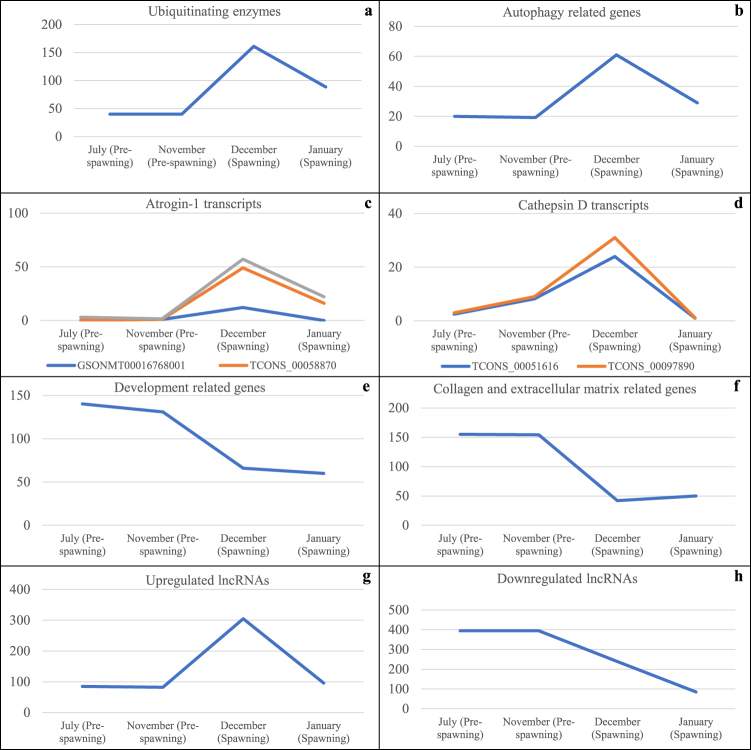
Transcript expression values of different classes of DE genes during pre-spawning and spawning months in atrophying muscle of diploid gravid fish: all ubiquitinating genes combined (**a**), all autophagy related genes combined (**b**), atrogin-1 transcripts (**c**), cathepsin D isoforms (**d**), all development related genes combined (**e**), all collagen and extracellular matrix related genes combined (**f**), all upregulated lncRNAs combined (**g**) and all downregulated lncRNAs combined (**h**). Expression level of each gene in gravid fish (2N) was normalized by expression level of respective gene in sterile fish (3N). Gene lists are provided in Supplementary dataset 2.

As fish progress from pre-spawning through spawning, severity of skeletal muscle atrophy increases as indicated by loss of muscle mass and muscle protein, and a reduction in muscle shear force,^28^ as a measure of ultrastructural changes associated with muscle breakdown. To further investigate the potential contribution of DE genes in sexual maturation associated muscle atrophy, we looked at the trends of expression pattern of DE genes over 4 months during pre-spawning (July, November) and spawning (December and January) using RNA sequencing data from our previously described source^3^. Transcript abundance of ubiquitin-proteasome system genes and autophagy-related proteolytic genes remained constant in July and November, sharply increased in December, and then declined in January (Fig. 3a,b). Expression level of proteases positively correlated with severity of muscle atrophy in the aforementioned timeframes. Late December represents the time of peak sexual maturation associated muscle atrophy. Expression of genes coding for different atrogin-1 isoforms and cathepsin D was highest in December and then declined in January (Fig. 3c,d). Cathepsin D is involved in sexual maturation associated muscle atrophy, and we found an increase in Cathepsin D transcript level. Nonetheless, previously we did not observe a significant change in catalytic activity of cathepsin D during atrophy^28^. Extracellular matrix protein genes and development related genes showed opposite expression trends (Fig. 3e,f), consistent with the loss of muscle firmness and development during atrophy. These findings suggest that DE genes may serve as reliable candidate(s) critical to sexual maturation associated muscle atrophy in fish. However, the trends of the gene expression showed in Fig. 3 should be taken with caution because a single RNA-Sea library from 10 pooled fish at each time point was used and no replicates were available to run statistical analyses”.

### Long noncoding RNAs (LncRNAs)

For differential lncRNA expression, we initially performed genome-wide discovery of lncRNA transcripts using RNA-Seq reads sequenced from skeletal muscle of gravid and sterile fish. Approximately 15,000 lncRNA transcripts were identified from this assembly and merged with our previously published lncRNA reference^32^; and both sources were used as a reference for gene expression analysis. A total of 1,160 lncRNAs were DE between atrophying and non-atrophying skeletal muscle. Of 1,160 DE lncRNAs, 225 and 10 lncRNAs had sequence homology with lncRNAs from Atlantic salmon and zebrafish, respectively (sequence identity: >80%, E value: <E-10, query cover: >50 nucleotides) (Supplementary dataset 3), but their functional annotation was not available in any species. Like protein-coding mRNAs, expression level of DE lncRNAs correlated with the severity of muscle atrophy during pre-spawning and spawning. Transcript abundance of upregulated lncRNAs remained constant during pre-spawning, but drastically increased in December and then declined in January (Fig. 3g). On the other hand, transcript abundance of downregulated lncRNAs showed an opposite trend (Fig. 3h). These findings suggest that expression of these DE lncRNAs may be involved in sexual maturation associated muscle atrophy in rainbow trout.

### MicroRNAs

A total of 28 microRNAs were DE between skeletal muscle of gravid and sterile fish. Of them, differential expression of mir-1, mir-133, and mir-29 during mammalian muscle atrophy has been previously reported^11,33^, but the remainder of DE microRNAs was reported for the first time (Table 1). A total of 665 unique mRNA genes were predicted as potential target genes of these 28 DE microRNAs; of these mRNA genes, 17 were also DE. Some of these DE microRNAs and their predicted DE mRNA targets showed reciprocal differential expression (Supplementary dataset 4). As an example, mir-29a predicted target mRNAs encoding collagen alpha, ATP binding cassette subfamily f member 3, alanine tRNA synthase, scavenger receptor class b member 1, and fk506-binding protein 2 were downregulated while mir-29a was upregulated in atrophying muscle. Similarly, mir-125b-1 was downregulated, and its predicted mRNA targets encoding CCAAT enhancer-binding protein delta and pancreatic progenitor cell differentiation and proliferation factor A were upregulated. Out of the DE microRNAs, sixteen were downregulated microRNAs, potentially targeting a total of 206 different protein-coding genes. Twenty-six of the predicted target genes were proteolytic enzymes, including genes involved in ubiquitin-proteasome and autophagy-lysosome mediated proteolysis (Supplementary dataset 4). Twelve upregulated microRNAs were predicted to target 468 different protein-coding genes. Consistent with their upregulated expression during atrophy, 101 predicted target genes were directly involved in extracellular matrix, muscle structure, or development (Supplementary dataset 4). Aforementioned findings suggest that some genes involved in muscle atrophy may not be necessarily regulated at transcription level, and its fate is determined post-transcriptionally by regulated expression of microRNA.

Let-7j was the most highly downregulated microRNA (−1056 × fold) in atrophying muscle (Table 1). It was predicted to target 63 different protein-coding genes that account for a wide range of functions (Supplementary dataset 4). Consistent with its downregulation in atrophying muscle, some of its predicted targets were proteolytic genes such as E3 ubiquitin-protein ligase NRDP1, ubiquitin-conjugating enzyme E2 and protein VPRBP (Supplementary dataset 4). Conversely, six different isoforms of mir-29 were highly upregulated in atrophying muscle (Table 1). Mir-29a, the most highly upregulated microRNA in atrophying muscle (11.7 × fold) was predicted to target 78 genes; the highest number of predicted target genes among DE microRNAs (Supplementary dataset 4). Consistent with its upregulation in atrophying muscle, predicted target genes of mir-29a included genes involved in muscle differentiation (IGF-BP 5), muscle sarcomere (e. g. myosin) structure, extracellular matrix (e. g. collagen), fat biosynthesis (e. g. long-chain-fatty-acid–ligase acsbg2 and acyl-coenzyme a thioesterase 11), protein synthesis (e. g. 60 s ribosomal protein l7) and development (e. g. prospero homeobox protein 1). These findings suggest that DE microRNAs may contribute to muscle atrophy by regulating proteolysis and other genes during muscle atrophy.

### Tissue specific and temporal expression of DE lncRNAs and mRNAs

**Figure 4:**
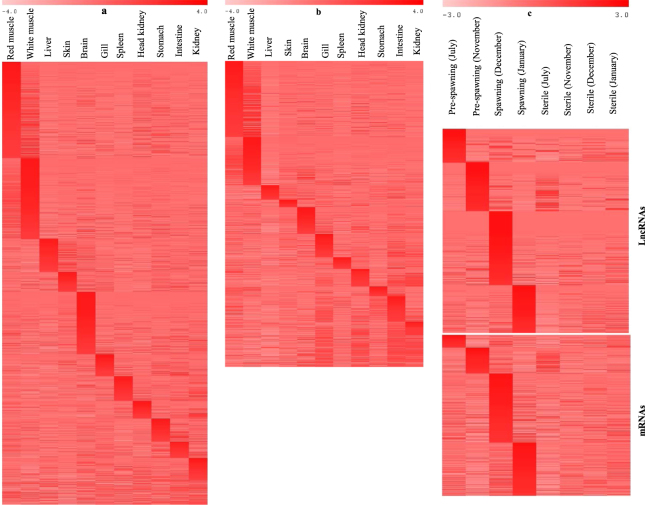
Heat map showing tissue specific expression pattern of DE lncRNAs (**a**), tissue specific expression pattern of DE mRNAs (**b**) and temporal expression pattern of DE lncRNAs and DE mRNAs during pre-spawning and spawning months (**c**). Value of color limit represents normalized expression values (Z scores). Darker red and lighter red colors represent higher and lower level of expression, respectively.

LncRNAs show strict spatial (tissue specific) and temporal (time dependent) expression patterns^34^. To investigate tissue specificity of DE genes, we studied their expression pattern across 13 vital tissues including red and white muscle (see method section for classification of tissue specific genes). About 40% (462/1,160) of DE lncRNAs and approximately 41% (348/852) of DE mRNAs were ‘specific’ to red or white muscle (Fig. 4). These specificities indicated about 2.5-fold enrichment of muscle specific lncRNAs, and about 5.5-fold enrichment of muscle specific mRNAs in the DE gene list compared to muscle specific expression of approximately 16% (8,460/51,644) of non-DE lncRNAs and approximately 6% (4,583/61,412) of non-DE mRNAs in the trout genome (Supplementary dataset 5). Interestingly, a majority of the most highly upregulated mRNAs were muscle ‘specific’. As an example, 47 out of 61 mRNAs, with fold change greater than 15, had muscle restricted expression patterns that included mRNA encoding atrogin-1. Muscle specific expression of atrogin-1 has also been previously reported in mammals^31^. In addition, some muscle specific lncRNAs, such as linc-MD1, are known to play an important role in regulation of muscle specific genes^18^. In addition to tissue specific expression, ~38% (442/1,160) of DE lncRNAs and ~40% (342/852) of DE mRNAs exhibited temporal (time dependent) expression patterns during pre-spawning and spawning (Fig. 4). These findings suggest that a significant proportion of the DE transcriptome in atrophying muscle is comprised of muscle specific gene expression, exhibiting a responsive expression pattern of muscle atrophy during sexual maturation.

### Genomic co-localization of DE lncRNA and mRNA genes

Functionally related lncRNAs and mRNAs physically co-localize in the genome^35^. Out of 1,160 DE lncRNAs, 231 (~20%) unique lncRNAs were either overlapped or were neighbored (<50 kb) by DE mRNA genes. A total of 242 (~28%) unique DE mRNA genes overlapped or neighbored DE lncRNAs (Supplementary dataset 6, tab 1 and 2). These findings suggest that DE lncRNAs and mRNAs tend to co-localize or cluster together in the genome. However, mere physical proximity does not necessarily lead to functional links^36,37^. To test the functional significance of physical proximity, we computed expression correlation between all neighboring/overlapping lncRNA-mRNA genes. Out of 387 neighboring/overlapping DE lncRNAs-mRNAs pairs, ~36.2% (140) had strongly correlated expression patterns (R > 0.85) compared to ~16.7% (168,207) DE lncRNAs-mRNAs with expression correlation (R > 0.85) regardless of genomic co-localization (Supplementary dataset 6, tab 3). The difference was statistically significant (Chi square p-value < 0.001) suggesting that co-localized lncRNA and protein-coding genes tend to be more correlated in expression than genes with greater separation in the genome. The degree of expression correlation between lncRNAs and protein-coding genes, as a function of physical proximity, was weakly negative (R = −0.35, p value < 0.001) (Supplementary dataset 6, tab 4). Next, we investigated whether ‘strand orientation’ (sense or antisense) was correlated with ‘type of expression correlation’ (negative or positive), and found no significant correlation (Chi square p-value > 0.05) (Supplementary dataset 6, tab 5). These observations suggest that DE lncRNA and mRNA genes tend to co-localize or cluster together in the genome, and often show correlated expression pattern. Further, to investigate the potential mechanistic regulation of all neighboring/overlapping lncRNA-mRNA genes, we scanned promoter sequences of the lncRNA-mRNA for transcription factor (TF) binding *cis* regulatory motifs. The majority of gene pairs harbored common TF binding motifs in their promoters. Many of the TFs are known to be involved in muscle development (e. g. myoD, myogenin, c-Fos, c-Jun, NF-AT1, Smad3, NF-Y, and NFI/CTF and 24 overlapped/neighboring lnc-mRNA pairs had ER-alpha in their promoters; Supplementary dataset 6, tab 6). Taken together, harboring the same TFs in the promoter regions, may partly, explain the correlated gene expression patterns of the co-localized DE lncRNA-mRNA pairs.

### DE lncRNAs acting as microRNA sponges or microRNA precursors

Direct ‘lncRNA-microRNA’ binding has important functional consequences including lncRNA mediated sponging of microRNA and ‘lncRNA-microRNA’ competition for mRNA binding^16^. To identify DE lncRNAs that potentially interfere with microRNA mediated gene regulation, we searched for high confidence microRNA binding sites in DE lncRNAs and mRNAs. A total of 134 trout microRNAs had binding sites in DE lncRNAs as well as DE mRNAs (Supplementary dataset 7). Some of these microRNAs, such as mir-133, mir-214, and mir-221, are known to regulate muscle atrophy or proteolysis^38,39^. DE lncRNAs shared microRNA binding sites with mRNAs encoding important proteolytic proteins such as atrogin-1, cathepsins, serine proteases, and several enzymes in the ubiquitin proteasome system (Table 3 and Supplementary dataset 7). For example, atrogin-1 (GSONMT00016768001), the most highly upregulated gene in atrophying muscle, shared mir-22-3p binding sites with Omy200063021 and Omy400145202. Expression patterns of a subset of LncRNAs and mRNAs that shared microRNA binding sites and were strongly correlated is shown in Table 3.

**Table 3:** DElncRNAs and mRNAs sharing microRNA binding sites and their expression correlation.

MicroRNA	DE mRNA with microRNA binding site (ID)	DE mRNA with microRNA binding site (Gene)	DE lncRNA with microRNA binding site	mRNA-lncRNA correlation (R)
omy-mir-22-3p	GSONMT00016768001	f-box only protein 32	Omy400145202	0.965
omy-mir-877-3p like	GSONMT00062643001	large neutral amino acids transporter small subunit 4-like	Omy400008946	0.998
hsa-miR-5007-5p like	GSONMT00070874001	insulin-induced gene 1	Omy400181081	0.991
eca-miR-9140 like	GSONMT00050732001	sestrin-1-like isoform x1	Omy400011543	0.985
aly-miR4235 like	GSONMT00065334001	dual specificity protein phosphatase 22-b-like	Omy400105663	0.985
hsa-miR-372-5p like	GSONMT00042478001	ring finger protein 122-like	Omy400004525	0.982
cfa-miR-8844 like	GSONMT00079999001	calcium-binding and coiled-coil domain-containing protein 1-like	Omy400016065	0.981
bta-miR-7865 like	GSONMT00005406001	ankyrin repeat and socs box protein 2-like isoform x2	Omy200187283	0.979
mml-miR-7189-3p like	GSONMT00018181001	alanine aminotransferase 2-like	Omy400068350	0.969
pma-miR-192-3p like	GSONMT00026025001	protein slowmo homolog 2-like	Omy200145928	0.968
pma-miR-192-3p like	GSONMT00026025001	protein slowmo homolog 2-like	Omy200145928	0.968
sbi-miR6219-5p like	GSONMT00004372001	transcriptional activator protein pur-beta-like	Omy100114534	−0.862
hsa-miR-486-5p like	GSONMT00015752001	complement c1q tumor necrosis factor-related protein 1-like	Omy500073247	−0.862
gga-miR-1606 like	GSONMT00079310001	s-adenosylmethionine synthase isoform type-2	Omy500028713	−0.835
bta-miR-6529a like	GSONMT00005466001	mitochondrial glutamate carrier 1-like	Omy500079466	−0.835
pma-miR-7a-3p like	GSONMT00031137001	c20orf24 homolog	Omy100068054	−0.832
bta-miR-7865 like	GSONMT00063472001	ras-related protein rab-7a	Omy500084871	−0.824
hsa-miR-5582-5p like	GSONMT00018534001	atp-binding cassette sub-family f member 3	Omy400105663	−0.821
cel-miR-1822-3p like	GSONMT00029001001	class e basic helix-loop-helix protein 40-like	Omy400025747	−0.805
mmu-miR-7029-5p like	GSONMT00081204001	homer protein homolog 1-like isoform x1	Omy500046047	−0.804

In addition to acting as a microRNA sponge, some lncRNAs serve as precursors of microRNAs and other classes of small noncoding RNA (sRNAs)^34,40^. DE lncRNA, Omy400148395, harbored mir-27 loci, and Omy200105075 harbored aly-miR-398c-like loci. Expression of mir-27 and aly-miR-398c-like microRNAs was positively correlated with their potential host lncRNAs; correlation R values were 0.60 and 0.81, respectively. This observation suggests that these microRNAs could be generated by post-transcription processing of lncRNA transcripts. Together, these findings suggest that some DE lncRNAs may sequester or generate microRNAs involved in muscle atrophy.

### Physical interaction between lncRNAs and protein-coding genes

Direct ‘lncRNA-mRNA’ physical interactions lead to mRNA decay^20^ and translation suppression^21^. To investigate potential existence of lncRNA-mRNA physical interactions, we used the IntaRNA tool that considers site accessibility and user defined seed requirement to predict the interaction^41^. At an interaction energy threshold <−100 Kcal/mole, 1,151 DE lncRNA-DE mRNA pairs exhibited potential physical interactions (Table 4 and Supplementary dataset 8). Interestingly, lncRNA-mRNA pairs, showing evidence of physical interaction, were more strongly correlated in expression (R > 0.85) than lncRNA-mRNA pairs without the potential physical interaction (Table 4 and Supplementary dataset 8).

**Table 4:** Potential physical interaction of DE lncRNA-DE mRNA and their expression correlation (top).

DE LncRNA	DE mRNA	LncRNA-mRNA hybrid length (nts)	Interaction energy (Kcal/mole)	Expression correlation (R^2^)
Omy500018678	f-box protein 32/atrogin-1	149	−229.635	0.92
Omy400015745	f-box only protein 32/atrogin-1	147	−144.674	0.90
Omy400044636	cathepsin D	147	−116.681	0.93
Omy400071240	ubiquitin carboxyl-terminal hydrolase 25-like	149	−170.963	0.90
Omy500080545	ubiquitin carboxyl-terminal hydrolase 10	146	−191.917	0.85
Omy500030058	collagen alpha-1 chain like	149	−240.644	0.88
Omy400028182	myosin-binding protein slow-type-like	128	−107.449	0.83
Omy400055397	ATP-dependent 6-phosphofructokinase	149	−148.776	0.82
Omy500034918	dnaJ homolog subfamily B member 1-like	150	−206.625	0.82
Omy500043112	serine threonine-protein kinase ulk2-like	149	−166.14	0.98
**DE lncRNA**	**DE protein**	**Interaction strength (%)**	**Discriminative power (%)**	**Expression correlation (R** ^**2**^ **)**
Omy400034255	f-box only protein 32/atrogin-1	100	96	0.57
Omy100083321	cathepsin L1	99	97	0.97
Omy400034255	cathepsin z precursor	99	97	0.94
Omy500058188	ubiquitin carboxyl-terminal hydrolase 10	100	100	0.89
Omy100109323	e3 ubiquitin-protein ligase trim63-like	98	97	0.98
Omy400181081	collagen alpha-1 chain-like	100	99	0.91
Omy400066578	autophagy-related protein 2 homolog a-like	99	96	−0.82
Omy500048471	autophagy-related protein 9a-like	99	98	0.91
Omy400025350	cyclic amp-dependent transcription factor atf-5	99	96	0.93
Omy200129177	kelch-like protein 38-like	100	100	0.93
Omy400015874	camp-responsive element modulator isoform	100	96	0.90
Omy400044055	ccaat enhancer-binding protein delta	100	96	0.76

Potential physical interaction between DE lncRNA and proteins of DE mRNAs (bottom).

In addition to interacting with mRNAs, several lncRNAs showed evidence of physical interaction with proteins of DE mRNAs. ‘LncRNA-protein’ physical interaction was computed using CatRapid Omics tool^42^. A total of 14,602 ‘DE lncRNA- protein’ pairs showed evidence of physical interaction at an interaction strength ≥96% and a discriminative power ≥96% (Table 4 and Supplementary dataset 8). Interestingly, ‘DE lncRNA-DE protein’ pairs showing evidence of direct physical interactions were more strongly correlated in expression (at transcript level) (R > 0.85) than ‘DE lncRNA-DE protein-coding gene’ pairs without the evidence of physical interaction (Chi square p-value < 0.001). Atrogin-1, cathepsin, and several enzymes of the ubiquitin-proteasome system were among the proteins with potential interaction with lncRNAs. ‘LncRNA-protein’ binding regulates protein’s stability^22^, availability^23^, activity^24^ and cellular localization^24^, suggesting that DE lncRNAs may have an important role in determining fate of DE protein-coding genes during muscle atrophy.

### ‘LncRNA-mRNA-microRNA’ interactome in atrophying muscle

**Figure 5:**
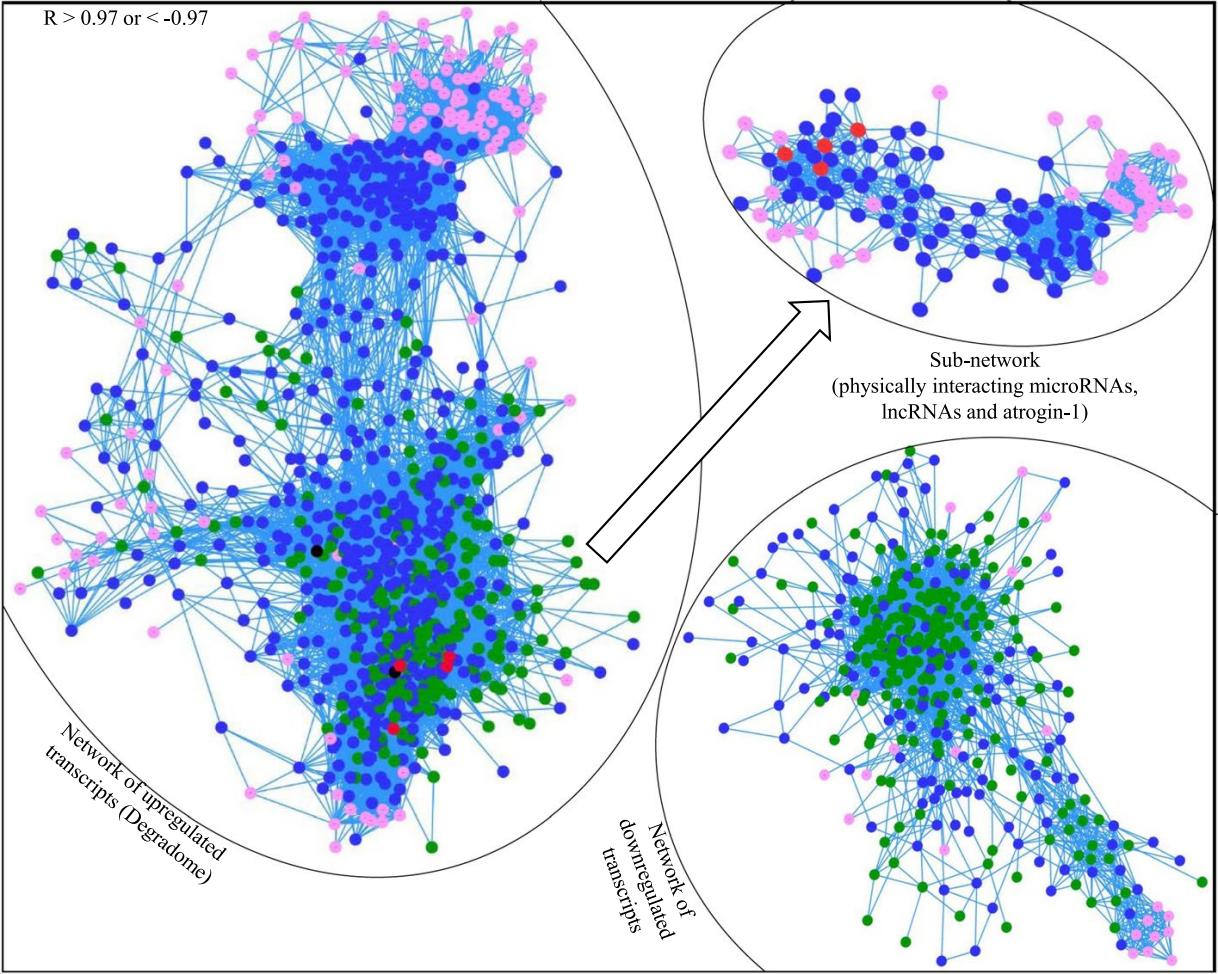
Gene expression network of DE lncRNAs (blue node), DE mRNAs (green node) and microRNAs (pink node) (R > 0.97 or <−0.97). Note that most of the DE genes are clustered in one of the two major networks. The larger network (degradome) comprises of upregulated genes and smaller network comprises of downregulated genes. In the network of upregulated transcripts, 4 atrogin-1 transcripts (red nodes) and mir-29 isoforms (black nodes) are in the center of the network. Sub-network drawn from the larger network contains 4 atrogin-1 transcripts, lncRNAs that bind to atrogin-1, and microRNAs that either bind to the lncRNAs and/or atrogin-1. Note: edges that connect nodes (genes) represent correlated expression at R cutoff 0.97 > or <−0.97; the shorter the length, the stronger the expression correlation.

LncRNAs, microRNAs, and mRNAs comprise interacted gene regulatory networks in the cell^43^, probably due to mutual regulation between microRNA and lncRNA^16^. To investigate existence of such gene interaction during muscle atrophy, we computed lncRNA-mRNA-microRNA interaction networks based on their expression pattern across 30 RNA-Seq datasets. At a correlation threshold R > 0.97 or <−0.97, about 50% (1,584) of DE transcripts were components of strongly correlated gene networks (Fig. 5). Interestingly, a majority of the correlated transcripts clustered in one of two major networks; the first comprised of downregulated transcripts and the second comprised of upregulated transcripts. The first network consisted of 430 transcripts (137 lncRNAs, 219 mRNAs and 74 microRNAs). The second network consisted of 960 transcripts (559 lncRNAs, 235 mRNAs and 166 microRNAs). The second network appeared to be an interacted gene “reactome” regulating muscle proteolysis because almost all upregulated proteolytic genes, including enzymes of ubiquitin proteasome system, autophagy related proteolytic genes and other proteases such as cathepsins, were in this network. Similarly, a majority of the upregulated microRNAs including mir-29, let-7, let-7d and mir-132 were in the network. Interestingly, atrogin-1 transcripts (the most highly upregulated transcripts) and mir-29a (the most highly upregulated microRNA) were in the center of this network suggesting a key role in the network. A crucial role of atrogin-1 in muscle atrophy has repeatedly been reported in fish and mammals^5,6,31^. We identified this network as ‘the rainbow trout muscle degradome’ because it appeared to be a regulatory network of muscle degrading coding and noncoding genes. The network was largely comprised of the genes that showed evidence of physical interaction with each other. A sub-network of the degradome shown in Fig. 5 consist of atrogin-1 transcripts, all DE lncRNAs that bind to atrogin-1 mRNAs, and microRNAs that either bind to lncRNAs and/or atrogin-1 mRNAs. The aforementioned observations suggest that coding and noncoding genes involved in muscle atrophy work in the form of a highly interacted gene network.

## Conclusion

Sexual maturation associated skeletal muscle atrophy serves as an excellent model to study piscine muscle proteolysis^1,3,29^. Previous efforts to investigate fish muscle proteolysis have provided limited information because these studies relied on individual or a limited set of protein-coding genes^1,5,6^. In the present study, we used deep lncRNA, mRNA, and microRNA sequencing approaches to investigate genes and gene regulatory networks that regulate muscle proteolysis in fish. Through investigation of the atrophying muscle transcriptome, we elucidated that fish muscle atrophy, like mammalian muscle atrophy, is regulated mainly by the ubiquitin-proteasome system. In addition, many autophagy-lysosomal proteases and transcription factors appeared to participate in muscle proteolysis during atrophication. Atrophying muscle exhibited upregulation of proteolytic genes with concomitant downregulation of genes involved in muscle sarcomere, extracellular matrix, protein and fat biosynthesis, and development. This trend in expression pattern of genes in atrophying muscle correlated well with characterizations of the atrophying muscle phenotype (e. g. muscle mass, protein content and muscle shear force), suggesting essential roles for DE genes in muscle atrophy. The present study identified a large number of new candidate coding and noncoding genes in addition to the genes identified by previous microarray and proteomic approaches^1,29^ suggesting that the RNA-Seq approach has identified a large number of reliable candidate genes involved in muscle proteolysis/degradation.

In present study, we characterized lncRNAs potentially involved in fish muscle proteolysis, and we investigated lncRNA-mRNA, lncRNA-microRNA and mRNA-microRNA interactions that potentially regulate muscle atrophy. A majority of DE lncRNAs and DE mRNA genes were co-localized in the genome and correlated in expression. DE lncRNAs appeared to physically interact extensively with DE protein-coding genes at transcript and protein levels. Similarly, DE lncRNA also showed potential to bind and sequester cellular microRNAs implicated in muscle proteolysis. LncRNA, mRNA and microRNAs that exhibited the aforementioned interactions are components of a highly correlated gene network in atrophying muscle. This finding indicates that the majority of genes involved in muscle proteolysis are expressed simultaneously by a common gene transcription program. Important to mention, ‘DE lncRNA-DE protein-coding’ gene pairs that either co-localized in the genome or showed evidence of direct physical interaction or competed for a common microRNA binding, were more frequently correlated in expression than random ‘DE lncRNA-DE protein-coding’ gene pairs. Perhaps this work is the first genome wide study that provides links between expression correlation and potential functional interactions between lncRNA and mRNA in fish. The present study has investigated potential coding and noncoding RNA interactions during muscle atrophy and contributes to our understanding of how energetic demand of sexual maturation triggers skeletal muscle atrophy in fish.

## Materials and Methods

### Ethics statement

Fish muscle tissues were obtained commercially from a private farm; therefore, Institutional Animal Care and Use Committee (IACUC) approval was not required.

### Fish population and muscle sampling

We previously described the fish population in another study^1^. Briefly, mature sterile (3N: triploid) and fertile (2N: diploid) female rainbow trout (about 500 gram) were obtained from Flowing Springs Trout Farm (Delray, WV) during spawning season; these fish were cultured in identical raceways. Water from a common spring was circulated in raceways at temperature 13 ± 3 °C. Both groups of fish were fed ad libitum (Zeiglar Gold; Zeigler Bros., Gardeners, PA) via demand feeder until sampling. At the time of muscle sampling gonado-somatic index (GSI) of fertile fish was 15.8 ± 0.3 (*n* = 5) compared to 0.3 ± 0.2 (*n* = 5) in sterile fish confirming the gravid stage of fertile fish. White muscle tissue of 8 fish (4 fertile and 4 sterile) was collected from the dorsal musculature, flash frozen in liquid nitrogen and stored at −80 °C until RNA extraction. Total RNA was extracted from the muscle using TRIzol method (Invitrogen, Carlsbad, CA). For phenotype measurement, boneless and skinless muscle fillet was obtained in a manual filleting procedure. Muscle yield was measured as percentage of whole body weight. A representative portion of the muscle fillet was used for shear force, pH, and proximate analyses (e.g. crude protein content, crude fat content, and moisture content).

### Library construction and sequencing

Sequencing libraries were prepared using Illumina TruSeq stranded total RNA with Ribo-Zero gold protocol following the manufacturer’s recommendations (Illumina Inc, CA, USA). One sequencing library was prepared from each fish and was provided a unique barcode. Equal amount of the barcoded libraries from all fish were pooled and sequenced using an Illumina HiSeq. 2000 sequencing platform in a single lane (2 × 100 reads). Similarly, for microRNA sequencing, Illumina’s TruSeq small RNA library preparation kit was used to prepare one barcoded library from each 8 fish and libraries were pooled and sequenced in a single lane of Illumina HiSeq. 2000 sequencing platform. Sequence data are available through the NCBI Sequence Read Archive (SRA) accession: SRP131630.

### Discovery of lncRNAs

LncRNAs from sequencing reads were identified by using the pipeline we described previously^32^. Briefly, reads were mapped to rainbow trout reference genome^44^ and assembled using TopHat and Cufflinks, respectively. Transcripts shorter than 200 nucleotides were filtered out. Protein-coding transcripts were removed by their sequence homology with NCBI protein entries. In addition, Coding Potential Calculator (CPC)^45^ tool was used to remove any transcripts with protein-coding potential (index value < −0.5). Other classes of noncoding RNAs were removed based on their sequence homology with noncoding RNA transcripts reported in public noncoding RNA databases including miRbase, genomic tRNA database, SSU (small subunit ribosomal RNA) and LSU (large subunit ribosomal RNA) databases. Putative lncRNA transcripts from the assembly are available at https://www.animalgenome.org/repository/pub/MTSU2017.1228/.

### Identification of DE mRNA, lncRNAs and microRNAs

Read mapping and identification of DE genes were performed using CLC genomics workbench. For protein-coding genes, sequencing reads from every fish were mapped to a mRNA reference from rainbow trout genome^44^ and our transcriptome assembly^46^. The expression value of each transcript was calculated in terms of TPM (transcript per million), and DE mRNAs between gravid and sterile fish were identified using EDGE test (FDR-P -value < 0.01, fold change: > 3 or <−3). For lncRNAs, previously published rainbow trout lncRNAs^32^ and additional lncRNAs assembled from this sequencing project were used as a reference. Read mapping and identification of DE lncRNA was done as described for mRNAs. For microRNAs, sequencing adapters were trimmed and reads were mapped to miRBase microRNA reference (release 21) (mismatch ≤ 2, additional/missing upstream/downstream bases ≤ 2). The total read count for each microRNA was calculated, and used to identify DE microRNAs by EDGE test (FDR-p-value < 0.01, fold change: > 3 or <−3).

### Real time PCR validation of DE transcripts

Total RNA from the same 8 fish used for sequencing was used to make template cDNA for qPCR analysis. Contaminating DNA in RNA sample was removed by DNAse treatment and cDNA was synthesized using Verso cDNA Synthesis Kit (Thermo Scientific, Hudson, NH). Transcript abundance of mRNA and lncRNA was quantified per manufacturer’s instruction using DyNAmo Flash SYBR Green Master Mix (Thermo Scientific, Hudson, NH) in Bio Rad CFX96™ System (Bio Rad, Hercules, CA). For microRNAs, miScript II RT kit (Qiagen, Valencia, CA, USA) was used to synthesize cDNA, and miScript^R^ SYBR^R^ green (Qiagen, Valencia, CA, USA)^47^ was used to quantify microRNA in Bio Rad CFX96™ System. The endogenous controls used for normalization were B-actin for mRNA and lncRNAs, and U6 for microRNA. None of the endogenous control genes was differentially expressed in this study. Fold changes in gene expression was calculated by using ΔΔCt method as described previously^35,48^. Mann-Whitney U test was used to check if the transcript level between atrophying and control muscle was statistically significant (p < 0.05). All 12 transcripts subjected to qPCR validation had Mann Whitney U test p-value < 0.05.

### Identification of tissue specific genes

The expression pattern of DE genes was investigated across 13 vital tissues: red muscle, white muscle, spleen, liver, skin, testis, brain, intestine, stomach, kidney, head kidney, gill and fat^44,46,49^. To identify tissue specific genes, we used a statistical approach described by^50^. The normalized expression value (z score) of every gene was calculated in each tissue from TPM counts. Each gene was classified as ‘specific’ to a tissue if z score was greater than 1.5 in that tissue and less than 1.5 in remaining 12 tissues which is similar to a previous study^50^. We used the same approach to identify genes ‘specific’ to a particular month during pre-spawning and spawning^3^, as described in the results section.

### LncRNA, mRNA and microRNA co-expression

For mRNA-ncRNA correlation analysis, 8 RNA-Seq samples from gravid and sterile fish and 22 RNA-Seq samples sequenced from muscle of selectively bred fish families developed at USDA/NCCCWA were used. Detailed description of these 22 samples, fish population, and sampling procedure has been previously described^51^. TPM values (for lncRNAs and mRNAs) and the total count (for microRNAs) were calculated in all 30 samples (8 samples from 2 ploidy groups and 22 samples from fish families) by mapping reads to corresponding mRNA, lncRNA, and microRNA references as described above. TPM and total count values were normalized by using a scaling method as previously described^52^, and normalized values were used to calculate expression correlation coefficients using Pearson correlations. A lncRNA-mRNA-microRNA expression network was constructed using Expression Correlation in cytoscape^53^.

### Identification of cis regulatory promoter motifs

Promoter regions of all neighboring/overlapping lncRNA-mRNA genes were scanned for putative Transcription factor (TF) binding motifs using ALGGEN PROMO TF motif search tool^54^. Maximum dissimilarity rate was set to 5%, and RE equality/query was set to  <0.05.

### Identification of microRNA-harboring lncRNAs

The rainbow trout genome reference^44^ was annotated with rainbow trout lncRNA reference sequences mentioned above. Rainbow trout pre-microRNA sequences (~64 nts long) from a recently published source^55^ were aligned to the lncRNA-annotated genome assembly. Pre-microRNA sequences that perfectly align (with no mismatch or gap) within annotated lncRNA loci in the genome were reported.

### LncRNA and mRNA targets of microRNAs

In the case of mRNAs, microRNA binding sites were searched in 3′ UTR; whereas for lncRNAs, target sites were searched for throughout the entire sequence length. Three target prediction algorithms: miRanda, PITA and TargetSpy were used to find target genes using sRNAtoolbox^56^. If the same target site is predicted by all 3 tools, it was considered as a potential microRNA target site. For all tools, minimum energy threshold was chosen as −20 Kcal/mole. Threshold scores chosen were 150 for miRanda and 0.99 for TargetSpy.

### Prediction of lncRNA-mRNA and lncRNA-protein interaction

LncRNA-mRNA direct physical interaction was predicted using IntaRNA-RNA-RNA interaction tool^41^. All lncRNA-mRNA interactions were recorded at an interaction energy threshold < −100 Kcal/mole. LncRNA-protein interactions were predicted using CatRapid Omics tool^42^. Interaction strength and discriminative power (a measure of predictability of interaction) were set at a ≥96% to consider lncRNA-protein interaction as putative interaction.

## Electronic supplementary material

Supplementary Dataset 1

Supplementary Dataset 2

Supplementary Dataset 3

Supplementary Dataset 4

Supplementary Dataset 5

Supplementary Dataset 6

Supplementary Dataset 7

Supplementary Dataset 8
