# Supplementary material for: Crosstalk among lncRNAs, microRNAs and mRNAs in the muscle ‘degradome’ of rainbow trout
Source: Sci Rep. 2018 May 30;8:8416. doi: 10.1038/s41598-018-26753-2 (PMC5976669; doi:10.1038/s41598-018-26753-2)

| **Crosstalk among lncRNAs, microRNAs and mRNAs in the muscle ‘degradome’ of rainbow trout** |
| --- |
| Bam Paneru, Ali Ali, Rafet Al-Tobasei, Brett Kenney and Mohamed Salem |


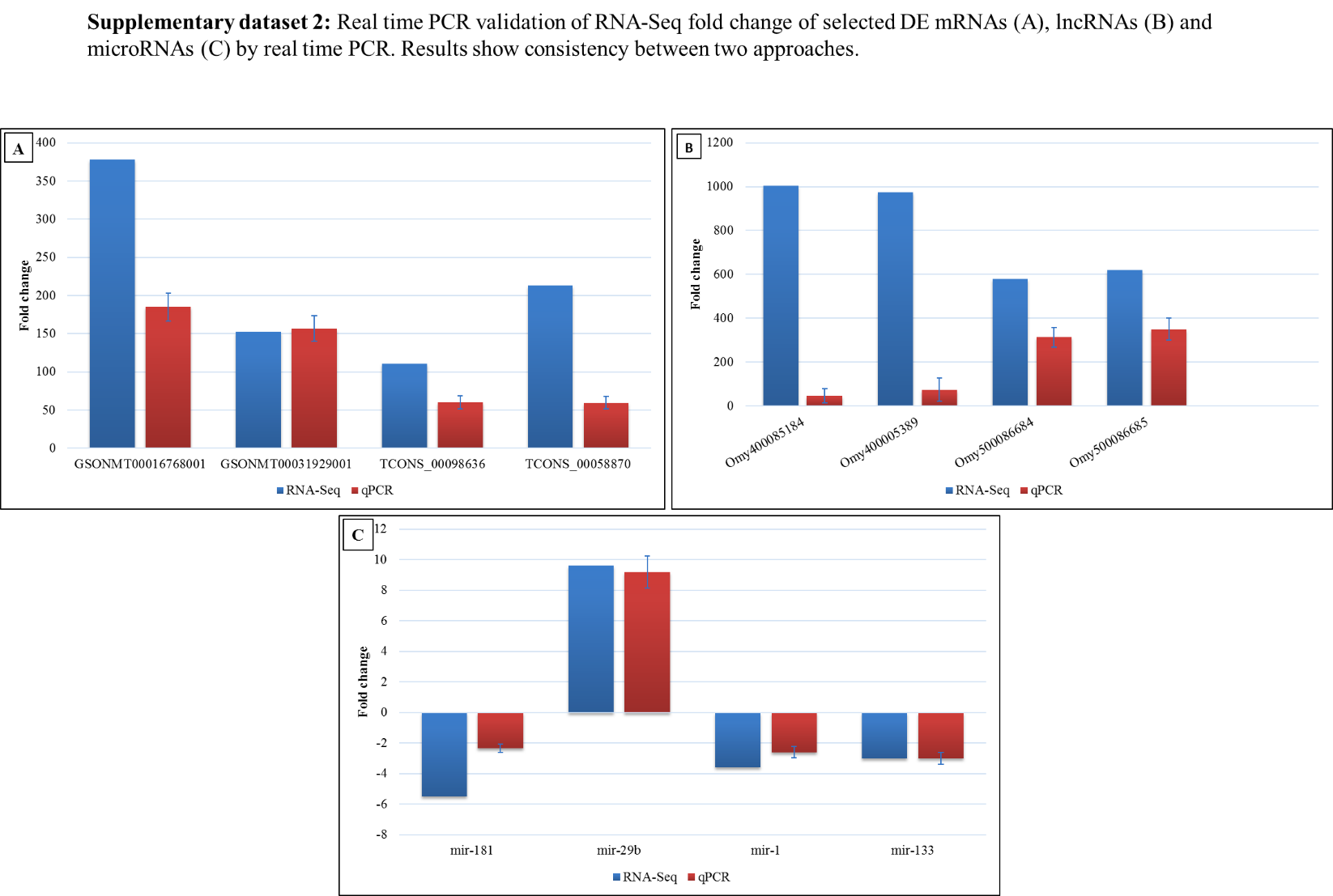

Supplement: Supplementary file 2 — Supplementary Dataset 2 [file 41598_2018_26753_MOESM2_ESM.docx]
